# Supplementary material for: The interactome of intact mitochondria by cross-linking mass spectrometry provides evidence for coexisting respiratory supercomplexes
Source: Mol Cell Proteomics. 2017 Dec 8;17(2):216–32. doi: 10.1074/mcp.RA117.000470 (PMC5795388; doi:10.1074/mcp.RA117.000470)
Supplement: Supplemental Data [file supp_RA117.000470_133922_0_supp_23978_fzffwf.zip › spectra_annotation/mito_DR_spectra_annotation/470-1-1-1-7-1.pdf]

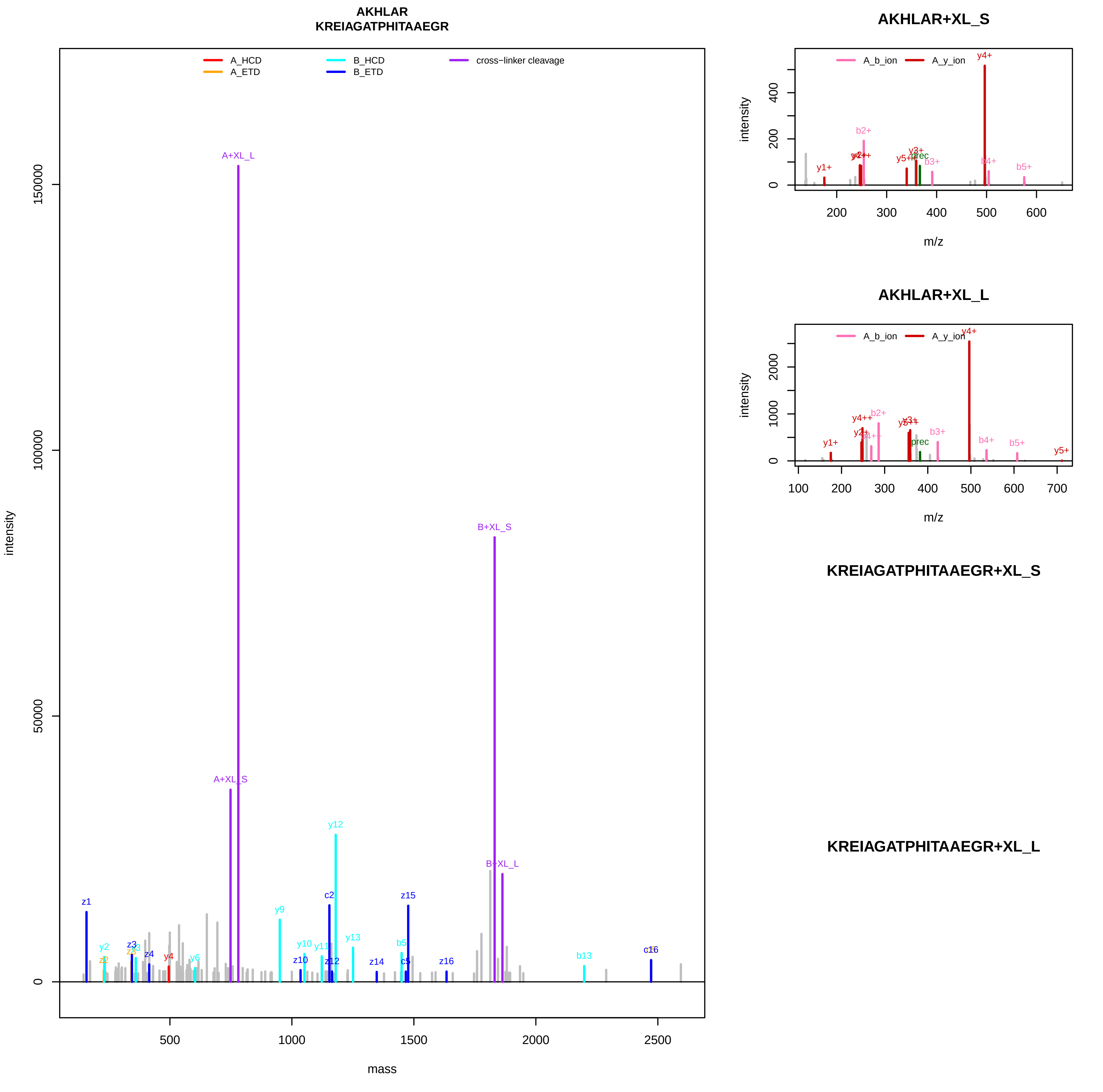
AKHLAR
KREIAGATPHITAAEGR
A_HCD
A_ETD
B_HCD
B_ETD
cross−linker cleavage
A+XL_L
A+XL_S
B+XL_S
B+XL_L
y12
y9
c2
z15
z1
y13
y10
y11
z12
z10
z14
z16
b5
c5
b13
c16
y2
z2
z3
z4
y4
y6
intensity
mass
0
50000
100000
150000
500
1000
1500
2000
2500
AKHLAR+XL_S
A_b_ion
A_y_ion
y4+
b2+
y1+
y3+
prec
y5++
b3+
b4+
b5+
intensity
m/z
0
200
400
300
500
600
AKHLAR+XL_L
A_b_ion
A_y_ion
y4+
y4++
b2+
y2+
y3+
y5++
y1+
prec
b3+
b4+
b5+
y5+
0
1000
2000
100
700
KREIAGATPHITAAEGR+XL_S
KREIAGATPHITAAEGR+XL_L
